# Preoperative 24-hour movement behaviors and early weight loss after metabolic bariatric surgery: a compositional analysis

**DOI:** 10.1038/s41366-025-01983-3

**Published:** 2025-12-12

**Authors:** Leah M. Schumacher, Yin Wu, J. Graham Thomas, Aurélie Baillot, Pavlos K. Papasavas, Sivamainthan Vithiananthan, Daniel B. Jones, Jennifer Webster, Dale S. Bond

**Affiliations:** 1 Department of Social and Behavioral Sciences, College of Public Health, Temple University, Philadelphia, PA USA; 2 Center for Obesity Research, Innovation, and Education, Digestive Health Institute, Hartford HealthCare, Hartford, CT USA; 3 Department of Psychiatry and Human Behavior, Warren Alpert Medical School of Brown University, Providence, RI USA; 4 Weight Control and Diabetes Research Center, The Miriam Hospital, Providence, RI USA; 5 École Interdisciplinaire de Santé, Université du Québec en Outaouais, Gatineau, QC Canada; 6 Institut du Savoir Montfort, Ottawa, ON Canada; 7 Centre de Recherche en Médecine Psychosociale, Centre Intégré de Santé et Services, Sociaux de L’Outaouais, Gatineau, QC Canada; 8 Cambridge Health Alliance, Cambridge, MA USA; 9 Harvard Medical School, Boston, MA USA; 10 Rutgers New Jersey Medical School, Newark, NJ USA

**Keywords:** Obesity, Weight management, Lifestyle modification

## Abstract

This study used compositional data techniques that address the interdependence of 24-h movement behaviors (sleep, sedentary behavior [SB], light-intensity physical activity [LPA], moderate-to-vigorous intensity physical activity [MVPA]) to examine: (1) how patients undergoing metabolic bariatric surgery (MBS) allocate time among these behaviors before MBS, and (2) whether overall time-use composition and modeled reallocation patterns relate to early weight loss after MBS. Participants wore an accelerometer 24 h/day for 10 days before MBS to measure time in sleep, SB, LPA, and MVPA. Isotemporal substitution models estimated differences in 6-month post-MBS percentage total weight loss (%TWL) associated with reallocations of these pre-surgery movement behaviors. Forty-five participants provided valid data. Pre-MBS time-use composition was associated with %TWL (23.8 ± 5.1%; *F* = 2.66, *p* = 0.047). Reallocating 15–60 SB or LPA minutes/day to MVPA was estimated to relate to 0.9–3.5% greater %TWL. Reallocating 15–30 MVPA minutes/day to SB or LPA was estimated to relate to 1.4–5.0% less %TWL (all comparisons *p* < 0.05). Other reallocations were non-significant. In conclusion, modeled shifts in time from SB or LPA to MVPA and vice versa were associated with estimated increases or decreases in early post-surgical weight loss, respectively. Experimental research is needed to clarify causal relationships and inform interventions to improve MBS outcomes.

## Introduction

Higher levels of moderate-to-vigorous intensity physical activity (MVPA) before and following metabolic bariatric surgery (MBS) are associated with more favorable postoperative weight outcomes [1–3]. However, MVPA is a movement behavior that accounts for the smallest proportion of a 24-h day [1, 4]. Recent work suggests that other daily movement behaviors, including sedentary behavior (SB) and sleep, may also influence weight outcomes [2, 4, 5]. Additionally, for some individuals undergoing MBS, increasing light-intensity physical activity (LPA) may be more practical than MVPA given physical or other limitations [6]. These findings support the idea of shifting from an MVPA-focused view to a more holistic and inclusive 24-h movement perspective [7].

Several recent studies have examined multiple 24-h movement behaviors in relation to postoperative weight outcomes [2, 4]. However, they have used analytic approaches that treat these behaviors separately, rather than reflecting their codependence [7–9]. Overlooking this codependence hinders understanding of how each 24-h movement behavior relates to weight outcomes in the context of the others [8–10]. Such approaches also do not address how reallocating time spent in one behavior (e.g., SB) to others (e.g., LPA) may influence weight outcomes [8–10].

To address these gaps, this study used compositional data analysis (CoDA) to 1) describe how patients allocate their time among sleep, SB, light-intensity PA (LPA), and MVPA before MBS from a 24-h perspective; 2) examine whether observed pre-MBS time-use composition correlates with weight loss at 6-months post-MBS; and 3) estimate differences in 6-month weight loss based on modeled compositional time reallocations among these movement behaviors pre-MBS. Although these time reallocations (Aim 3) were statistically modeled rather than experimentally tested, they can offer insights into how shifts in 24-h time use might influence weight loss and inform further experimental investigation. We hypothesized that overall time-use composition and reallocating time away from SB and LPA to MVPA would be associated with greater weight loss, while the reciprocal reallocation pattern would be associated with less weight loss.

## Methods

Data were collected as part of a larger study investigating psychosocial and behavioral predictors of post-MBS weight loss (R01DK108579) [11]. All protocol elements relevant to the current study are reported below. All procedures were approved by The Miriam Hospital Institutional Review Board (#2112).

### Participants

Participants were recruited from two academic MBS clinics in the Northeastern United States between May 2016 and April 2018. Participants were ≥21 years old, had a body mass index (BMI) ≥ 35.0 kg/m^2^, and were scheduled to undergo Roux-en-Y gastric bypass (RYGB) or sleeve gastrectomy (SG)—the two most common MBS procedures [12]. Additional details on inclusion and exclusion criteria, as well as recruitment and screening procedures, have been previously reported [11].

### Procedures

After eligibility was confirmed and informed consent was obtained, participants completed an assessment visit 3–8 weeks before their scheduled MBS procedure. During this visit, they received an Actigraph GT9X accelerometer to wear on their non-dominant wrist 24 h/day for 10 days, completed a sociodemographic questionnaire, and had their height and weight measured. Participants’ height and weight were re-measured by clinic staff at 6 months post-MBS. Participants received USD75 compensation for each assessment.

### Measures

Actigraph data were processed using GGIR, a commonly used R package for processing accelerometry data [13]. Participants who wore the device for ≥16 h/day on ≥4 days were included in the analysis [13]. Time/day spent in sleep was determined using the vanHees2025 algorithm [14] for sustained inactivity bouts detection, and the Heuristic algorithm looking at Distribution of Change in Z-Angle (HDCZA) algorithm [15] as the guider. For awake hours, vector magnitude counts per minute thresholds were used to identify time/day spent in SB, LPA, and MVPA as follows: <2000 counts/min = SB, 2000–7499 counts/min = LPA and ≥7500 counts/min = MVPA [16, 17].

Participants self-reported their age, gender, race, ethnicity, and MBS procedure type. Percent total weight loss (%TWL) was calculated using pre- and 6-month post-MBS weights.

### Statistical analysis

Aim 1 was evaluated using descriptive statistics (mean and standard deviation). For Aim 2, Compositional Data Analysis (CoDA) was used to perform linear regression models on the 6-month %TWL while allowing the 24-h movement behaviors composition (i.e., using the relative values of time/day between behaviors which sum up to 1 representing 1440 min) before MBS to be included as one of the model predictors. In the same linear regression model, age, sex, race, pre-MBS BMI, and surgery type were each evaluated as potential covariates and adjusted for if significant. The four movement behaviors (sleep, SB, LPA, MVPA) in the composition were transformed to four sets of three isometric log ratio (ILRs) coordinates, using the *compositions* package [18]. Of note, the relative time spent in each of the four 24-h movement behaviors as a composition is represented by a 4-part simplex, which is not compatible with operations suitable for real space (e.g., multiplication). By using ILR coordinates, the 4-part simplex can be structured as a 3-dimensional real space, while the relative positions of the data points are preserved from the simplex to the real space (i.e., isometric). Data represented by the new 3-dimensional real space is then compatible with conventional statistical models such as multiple linear regression [19]. If the overall composition (ILRs’ coordinates) was significantly associated with %TWL (*p* < 0.05), isometric time reallocation analyses were conducted (Aim 3), using the codaredistlm package [20, 21], to estimate the average difference in 6-month %TWL when time (−60 to +60 min at 15 min increments) in one behavior at pre-MBS was replaced in the model by time in another behavior at pre-MBS, while keeping all remaining behaviors constant. All analyses were performed in R version 4.4.1. Statistical significance was set at α = 0.05.

## Results

Forty-five (90%) participants had 6-month weight loss data and sufficient baseline accelerometer wear time. On average, participants were middle-aged (45.2 ± 11.3 years old) and had class III obesity before MBS (46.4 ± 7.7 kg/m^2^). Most identified as women (88.9%), White (59.1%), and non-Hispanic (82.2%). Nine (20.0%) patients underwent RYGB and 36 (80.0%) underwent SG. Surgery type was controlled for in the regression models. Other variables were not significant and were not included.

Aims 1 and 2. On average, participants had 447.3 ± 70.7 sleep, 583.7 ± 100.9 SB, 366.0 ± 75.3 LPA, and 42.6 ± 23.0 MVPA minutes/day at pre-MBS over 9.4 ± 2.4 days. Mean 6-month TWL was 23.8 ± 5.1%. Pre-MBS time-use composition was associated with 6-month %TWL (*F* = 2.66, *p* = 0.047).

**Fig. 1:**
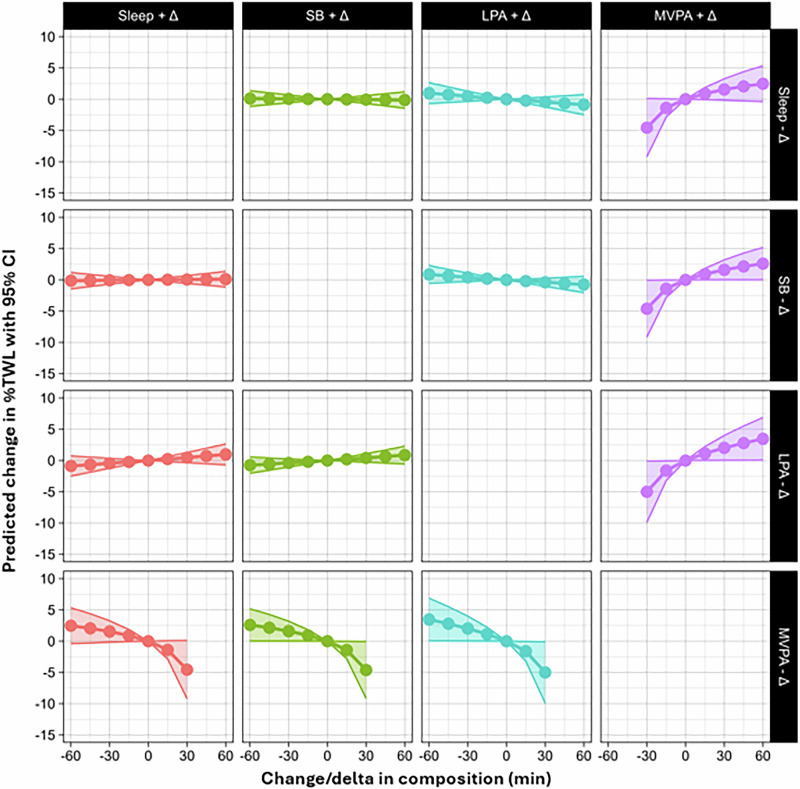
Estimated associations between changes in pre-MBS time-use and 6-month post-MBS weight loss. Each panel within the figure depicts a separate time-use reallocation model. The labels along the top show the 24-h time use behavior that was increased (reallocated to) for each model, and the labels along the right side show the time use behavior that was decreased (reallocated from) for each model. The x-axis at the bottom shows the number of minutes for each modeled reallocation. The y-axis on the left side shows estimated 6-month post-MBS percentage total weight loss. The plotted values within each panel show the estimated change in percentage total weight loss for each modeled reallocation with 95% confidence intervals. SB sedentary behavior, LPA light-intensity physical activity, MVPA moderate-to-vigorous intensity physical activity, TWL total weight loss.

Aim 3. As shown in Table 1 and Fig. 1, per the estimated models, shifting 15–60 min of SB or LPA to MVPA was associated with greater 6-month %TWL (all comparisons were statistically significant at *p* < 0.05). Conversely, shifting 15 or 30 min of MVPA to SB or LPA was associated with less %TWL (all comparisons *p* < 0.05). All other reallocations between movement behaviors and %TWL were statistically non-significant.

**Table 1:** Associations between various time use reallocations and 6-month total weight loss.

Time-use behavior displaced by	Time-use behavior increased by			
15 min	Sleep	SB	LPA	MVPA
Sleep	NA	−0.03 (−0.35, 0.29)	−0.23 (−0.63, 0.18)	0.90 (−0.08, 1.87)
SB	0.03 (−0.29, 0.35)	NA	−0.20 (−0.53, 0.14)	**0.92 (0.01, 1.84)**
LPA	0.23 (−0.18, 0.64)	0.20 (−0.14, 0.55)	NA	**1.13 (0.02, 2.23)**
MVPA	−1.4 (−2.86, 0.07)	**−1.42 (−2.83, −0.02)**	**−1.62 (−3.21, −0.03)**	NA
30 min	Sleep	SB	LPA	MVPA
Sleep	NA	−0.06 (−0.71, 0.59)	−0.45 (−1.26, 0.37)	1.55 (−0.17, 3.27)
SB	0.06 (−0.58, 0.69)	NA	−0.39 (−1.05, 0.28)	**1.61 (0.02, 3.20)**
LPA	0.47 (−0.35, 1.30)	0.41 (−0.28, 1.11)	NA	**2.03 (0.04, 4.01)**
MVPA	−4.56 (−9.22, 0.11)	**−4.61 (−9.18, −0.05)**	**−5.00 (−9.91, −0.08)**	NA
45 min	Sleep	SB	LPA	MVPA
Sleep	NA	−0.10 (−1.08, 0.89)	−0.66 (−1.88, 0.55)	2.06 (−0.28, 4.39)
SB	0.08 (−0.87, 1.03)	NA	−0.57 (−1.55, 0.42)	**2.15 (0.02, 4.28)**
LPA	0.72 (−0.53, 1.97)	0.64 (−0.42, 1.69)	NA	**2.79 (0.06, 5.52)**
MVPA	–	–	–	NA
60 min	Sleep	SB	LPA	MVPA
Sleep	NA	−0.13 (−1.45, 1.19)	−0.87 (−2.49, 0.75)	2.47 (−0.39, 5.34)
SB	0.10 (−1.16, 1.37)	NA	−0.75 (−2.05, 0.56)	**2.60 (0.02, 5.17)**
LPA	0.97 (−0.70, 2.65)	0.87 (−0.56, 2.29)	NA	**3.47 (0.06, 6.88)**
MVPA	–	–	–	NA

Numbers are estimated change values (95% CI) in percent of total weight loss (%TWL) corresponding to the modeled reallocation of time. Positive values indicate greater magnitude of weight loss, negative values indicate smaller magnitude of weight loss. Bolded values were statistically significant. – indicate the magnitude of time reallocation exceeded the allowed amount and resulted in values that were not sensible.

*SB* time in sedentary behavior, *LPA* light intensity physical activity, *MVPA* moderate to vigorous intensity physical activity.

## Discussion

This study is the first to use compositional data analysis to evaluate whether time spent in movement behaviors across a 24-h day before MBS relates to early postoperative weight loss. Unlike earlier studies that focused on individual 24-h movement behaviors and associations with weight loss without considering the proportional time spent in other behaviors [1–5], we found that the overall 24-h composition of time in sleep, SB, LPA, and MVPA before MBS was associated with weight loss afterward. Additionally, our findings indicated that certain modeled time reallocations among these behaviors were associated with estimated differences in weight loss at 6-month post-MBS.

Reallocating 15–60 min per day from SB or LPA to MVPA before MBS was estimated (modeled) to relate to greater 6-month postoperative weight loss (up to 3.5% greater %TWL), while reallocating time away from MVPA was estimated to relate to less 6-month postoperative weight loss (up to 5.0% less %TWL). Although these results do not reflect experimental manipulation of these movement behaviors, they suggest the need for further research to determine whether even modest shifts in time-use patterns—such as replacing 15 min of SB or LPA with MVPA, which may be more feasible in this population—could lead to clinically meaningful changes in postoperative weight loss.

Our findings align with recent research in nonsurgical samples, which shows meaningful changes in obesity and cardiometabolic health indicators with small actual or predicted time reallocations (e.g., 10 min), with more pronounced benefits with larger shifts [21, 22, 23]. It is noteworthy that replacing MVPA with lower-intensity behaviors was estimated to have a stronger, negative association with weight change; for example, replacing 30 min of MVPA with LPA was associated with an estimated 5% less %TWL. These findings suggest that reallocating time to and especially away from MVPA before MBS may influence weight loss during the early postoperative period, when MBS has its greatest impact on body weight, although experimental verification is needed.

Results also showed that participants’ observed (measured), pre-surgical time-use patterns related to 6-month postoperative weight loss. The reasons why preoperative movement patterns relate to early postoperative weight loss are not yet fully understood. However, individuals with more favorable movement patterns may have higher total energy expenditure, be healthier overall, face lower risks of postoperative complications, and be more inclined to follow a healthier lifestyle that promotes more weight loss after MBS. Additional research is needed to examine these possibilities while accounting for potential confounders, as well as to explore how shifts in time-use composition and movement behaviors relate to other clinical- and patient-centered outcomes.

Study strengths include device-based measurement of 24-h movement behaviors, calculation of 24-h time-use patterns from multiple days of data, rigorous statistical methods that account for the interdependence of these behaviors, measured weight, and a diverse sample. However, certain limitations must be considered: our allocation analyses relied on estimated (statistically modeled) rather than observed effects; without experimental testing, it remains uncertain whether, for example, reallocating time from SB or LPA to MVPA before MBS indeed increases %TWL after MBS. The modest sample size also limited the investigation of changes in movement behaviors or their associated with %TWL beyond six months post-surgery. Moreover, this study did not assess causal pathways, potential mechanisms driving the observed relationships, eating behaviors or other factors that could influence weight loss after MBS, the role of 24-h movement behaviors in other clinical and patient-centered outcomes, or the context and other aspects of these behaviors (e.g., sleep quality or prolonged versus dispersed SB) that might affect weight loss and general health. Future work that addresses these gaps, including larger samples with more men, is needed to confirm these findings and improve generalizability.

In conclusion, this study is the first to examine how patients undergoing MBS allocate their time to different movement behaviors over the 24-h day before MBS using a compositional approach and to model how time-use changes may relate to postoperative weight loss. Both the overall, observed 24-h composition and the modeled reallocation of time to and from MVPA—including smaller increments (e.g., 15 min), which may be more achievable for this population—were associated with early weight loss after MBS. These findings highlight the importance of expanding beyond an exercise-focused perspective and adopting a holistic view of movement in the context of MBS to better understand how movement behaviors intersect and evolve over time, potentially enhancing and sustaining clinical and health outcomes.

## Data Availability

The datasets generated during and/or analyzed during the current study are available from the corresponding author on reasonable request.
